# Supplementary material for: COVID-19 vaccine hesitancy among women planning for pregnancy, pregnant or breastfeeding mothers in Jordan: A cross-sectional study
Source: PLoS One. 2023 Jun 1;18(6):e0286289. doi: 10.1371/journal.pone.0286289 (PMC10234543; doi:10.1371/journal.pone.0286289)
Supplement: S1 Table — (DOCX) [file pone.0286289.s001.docx]

**Table 1. Characteristics of participating women**

| **Variable** | **Mean ± SD or frequency (%)**  **N=874** |
| --- | --- |
| **Age** | 36.63 ± 10.18 |
| **Marital status:**  Single  Married  Divorced  Widowed | 348 (39.8%)  492 (56.3%)  24 (2.7%)  10 (1.1%) |
| **Are you:**  Planning for pregnancy  Pregnant women  Breastfeeding women  Others | 136 (15.6%)  210 (24.0%)  82 (9.4%)  446 (51.0%) |
| **COVID-19 vaccination:**  Not taken  One dose  Two doses  Three doses | 234 (26.8%)  178 (20.4%)  290 (33.2%)  172 (19.7%) |
| **Previous COVID-19 infection:**  Yes  No | 490 (56.1%)  384 (43.9%) |
| **Chronic diseases:**  No medical history  Hypertension  Diabetes mellitus  Immune system disease  Others | 696 (79.6%)  52 (5.9%)  56 (6.4%)  28 (3.2%)  42 (4.8) |
| **Employment:**  Employed  Unemployed | 510 (58.4%)  364 (41.6%) |
| **Residency:**  Amman  Zarqa  Irbid | 470 (53.8%)  247 (28.3%)  157 (18%) |
| **Level of education:**  Primary education  Secondary education  Bachelor  High education | 26 (3%)  48 (5.5%)  757 (86.6%)  43 (4.9%) |
| **Financial status:**  Comfortable  Varies  Tight | 210 (24.03%)  290 (33.18%)  374 (42.79%) |
| **Years of education** | 15.37 ± 2.71 |
