# Supplementary material for: COVID-19 vaccine hesitancy among women planning for pregnancy, pregnant or breastfeeding mothers in Jordan: A cross-sectional study
Source: PLoS One. 2023 Jun 1;18(6):e0286289. doi: 10.1371/journal.pone.0286289 (PMC10234543; doi:10.1371/journal.pone.0286289)
Supplement: S2 Table — (DOCX) [file pone.0286289.s002.docx]

**Table 2. Perception of the disease, vaccine hesitancy, benfits and motivation**

| Variable | **Mean ± SD** |
| --- | --- |
| **Perception of seriousness of COVID-19** | 8.78 ± 2.70 |
| **Vaccine hesitancy** | 26.59 ± 7.86 |
| **Perceived benefits of vaccine** | 11.24 ± 3.65 |
| **Motivation and cause of action of taking vaccine** | 8.94 ± 2.87 |
