# Supplementary material for: COVID-19 vaccine hesitancy among women planning for pregnancy, pregnant or breastfeeding mothers in Jordan: A cross-sectional study
Source: PLoS One. 2023 Jun 1;18(6):e0286289. doi: 10.1371/journal.pone.0286289 (PMC10234543; doi:10.1371/journal.pone.0286289)
Supplement: S3 Table — (DOCX) [file pone.0286289.s003.docx]

**Table 3. Differences in perceptions of the seriousness of COVID-19, vaccine hesitancy, perceived benefits of the vaccine, and motivation and causes of action about taking the vaccine according to pregnancy status of the women**

| **Dependent variable**  **Mean ± SD** | **Grouping variable: pregnancy status**  **Mean ± SD** | | **Mean difference** | **P value** | **F** |
| --- | --- | --- | --- | --- | --- |
| Perception  8.78 ± 2.70 | pregnant women 7.53 ± 1.80 | planning to be pregnant 7.12 ± 0.72 | 0.42 | .103 | 108.73 |
|  |  | breastfeeding women 7.24 ± 0.73 | 0.29 | .336 |  |
|  |  | Others 10.6 ± 2.94 | -2.62 | .000 |  |
|  | planning for pregnancy 7.12 ± 0.72 | breastfeeding women 7.24 ± 0.73 | -0.01 | .696 |  |
|  |  | Others 10.6 ± 2.94 | -3.04 | .000 |  |
|  | breastfeeding women 7.2439 ± 7296 | Others  10.1570 ± 2.9417 | -2.91 | .000 |  |
| Hesitancy  26.5881 ± 7.85957 | pregnant women 30.11 ± 4.49 | planning to be pregnant 31.32 ± 6.40 | -1.21 | .109 | 93.49 |
|  |  | breastfeeding women 30.27 ± 6.29 | -.154 | .863 |  |
|  |  | Others 22.81 ± 7.91 | 7.31 | .000 |  |
|  | planning for pregnancy  31.32 ± 6.40 | breastfeeding women 30.27 ± 6.29 | 1.06 | .271 |  |
|  |  | Others 22.81 ± 7.91 | 8.52 | .000 |  |
|  | breastfeeding women 30.27 ± 6.29 | Others 22.81 ± 7.91 | 7.46 | .000 |  |
| Benefits 11.2414 ± 3.55823 | pregnant women 8.73 ± 1.93 | planning to be pregnant 8.92 ± 2.15 | -0.19 | .522 | 221.33 |
|  |  | breastfeeding women 9.09 ± 2.10 | -0.36 | .304 |  |
|  |  | Others 13.53 ± 3.18 | -4.81 | .000 |  |
|  | planning for pregnancy 8.92 ± 2.15 | breastfeeding women 9.09 ± 2.10 | -0.17 | .651 |  |
|  |  | Others 13.53 ± 3.18 | -4.62 | .000 |  |
|  | breastfeeding women 9.09 ± 2.10 | Others 13.53 ± 3.18 | -4.45 | .000 |  |
| Motivation and cause of action 8.9405 ± 2.86722 | pregnant women 6.7524 ± 1.40 | planning to be pregnant 7.15 ± 1.71 | -0.39 | .092 | 240.86 |
|  |  | breastfeeding women 7.27 ± 1.68 | -0.52 | .062 |  |
|  |  | Others 10.83 ± 2.55 | -4.07 | .000 |  |
|  | planning for pregnancy 7.15 ± 1.71 | breastfeeding women 7.27 ± 1.68 | -0.12 | 683 |  |
|  |  | Others 10.83 ± 2.55 | -3.68 | .000 |  |
|  | breastfeeding women 7.27 ± 1.68 | Others 10.83 ± 2.55 | -3.56 | .000 |  |
