# Supplementary material for: COVID-19 vaccine hesitancy among women planning for pregnancy, pregnant or breastfeeding mothers in Jordan: A cross-sectional study
Source: PLoS One. 2023 Jun 1;18(6):e0286289. doi: 10.1371/journal.pone.0286289 (PMC10234543; doi:10.1371/journal.pone.0286289)
Supplement: S4 Table — (DOCX) [file pone.0286289.s004.docx]

**Table 4. Difference in the perception of COVID-19 seriousness, vaccine hesitancy, perceived benefits, and motivation and causes of action of taking the vaccine according to previous infection with COVID-19, history of chronic diseases and marital status**

| **Variable** | **Grouping variable** | | **Mean ± SD** | **P value** | **T** |
| --- | --- | --- | --- | --- | --- |
| Perception | Previous infection: | Yes (490) | 8.40 ± 2.56 | .000 | -4.754 |
|  |  | No (384) | 9.27 ± 2.81 |  |  |
| Hesitancy | Previous infection: | Yes (490) | 29.42 ± 6.82 | .000 | 3.192 |
|  |  | No (384) | 22.97 ± 7.61 |  |  |
| Benefits | Previous infection: | Yes (490) | 10.18 ± 3.30 | .000 | -10.534 |
|  |  | No (384) | 12.59 ± 3.42 |  |  |
| Motivation | Previous infection: | Yes (490) | 8.05 ± 2.65 | .000 | 11.027 |
|  |  | No (384) | 10.07 ± 2.74 |  |  |
| Perception | Chronic diseases: | Yes (178) | 8.03 ± 2.21 | .000 | -4.17 |
|  |  | No (696) | 8.97 ± 2.79 |  |  |
| Hesitancy | Chronic diseases: | Yes (178) | 28.60 ± 6.67 | .000 | 3.84 |
|  |  | No (696) | 26.07 ± 8.06 |  |  |
| Benefits | Chronic diseases: | Yes (178) | 7.38 ± 1.54 | .000 | -8.45 |
|  |  | No (696) | 9.30 ± 2.30 |  |  |
| Motivation | Chronic diseases: | Yes (178) | 9.42 ± 1.95 | .000 | -7.90 |
|  |  | No (696) | 11.71 ± 3. 72 |  |  |
| Perception | Marital status: | Married (492) | 7.19 ± 1.35 | .000 | 26.58 |
|  |  | Unmarried (382) | 10.83 ± 2.62 |  |  |
| Hesitancy | Marital status: | Married (492) | 30.11 ± 5.41 | .000 | -17.51 |
|  |  | Unmarried (382) | 22.04 ± 8.19 |  |  |
| Benefits | Marital status: | Married (492) | 9.17 ± 2.52 | .000 | 25.98 |
|  |  | Unmarried (382) | 13.91 ± 2.86 |  |  |
| Motivation | Marital status: | Married (492) | 7.24 ± 1.99 | .000 | 26.79 |
|  |  | Unmarried (382) | 11.13 ± 2.29 |  |  |
