# Supplementary material for: COVID-19 vaccine hesitancy among women planning for pregnancy, pregnant or breastfeeding mothers in Jordan: A cross-sectional study
Source: PLoS One. 2023 Jun 1;18(6):e0286289. doi: 10.1371/journal.pone.0286289 (PMC10234543; doi:10.1371/journal.pone.0286289)
Supplement: Attachment — Submitted filename: reviewer Plos. vaccine hesitancy.docx [file pone.0286289.s006.docx]

COVID-19 vaccine hesitancy among Jordanian women who are planning to be pregnant, are pregnant or are breastfeeding

This study assessed COVID-19 vaccine hesitancy among Jordanian women who are planning to be pregnant, are pregnant or are breastfeeding.

I did not see any scientific merit of the study. Indeed the analysis was simple but not strong

The researcher made assumptions that women did not receive information. You have to consider that this is a new pandemic and little evidence is available. So hesitancy is normal part of acceptance

Abstract: Clear, I wish to see assessment of whether they received knowledge about vaccine or not with regards to pregnancy. You do not know whether it will impact their decision. In your recommendations you are making assumptions but you never assessed that before your study. This is important to consider. Do you have any data on this variable? It will be great addition

Introduction: Clear and concise

Section 2.3. could you please verify or elaborate what do you mean by other women.

In the method

You have stated that “This questionnaire has been previously used in studies around the globe, and has been 7 reported to be a valid and reliable questionnaire to be used in Arabic communities (Lin et al., 2020, Samannodi, 2021).

The first references looks like in China???

LIN, Y., HU, Z., ZHAO, Q., ALIAS, H., DANAEE, M. & WONG, L. P. J. P. N. T. D. 2020. Understanding COVID-19 vaccine demand and hesitancy: A nationwide online survey in China. 14, e0008961

Please double check and replace with other Arabic based studies

Ethical considerations

Please add the IRB number

Data collection procedure

Why you will find breast feeding women and those who are planning to get pregnant in maternity clinics. Do not you think this is a limited site for recruitment

I suggest you make justification

In table 1. Move age first, usually age and gender at the beginning

Titles of all tables should be revised to include names of groups and N

In your results you have stated

Independent samples t-test was used to examine the difference in the perception of COVID19 seriousness, vaccine hesitancy, perceived benefits, and motivation and causes of action of taking the vaccine according to previous infection with COVID-19, history of chronic diseases, and marital status.

Why only those two variables?

I was expected to see multiple regression analysis at the end. Why you stopped your data at bivariate Pearson’s correlations. I think a stronger prediction model will be good addition

Also, I did not see other women in the analysis

You did anova and I was expected to see posthoc to include other women. ???

Discussion

The current study showed that women who were married, pregnant women, planning to be pregnant, or breastfeeding women had higher COVID-19 vaccination hesitancy compared to other women.

Why you added the word married. It is a given fact
