# Supplementary material for: COVID-19 vaccine hesitancy among women planning for pregnancy, pregnant or breastfeeding mothers in Jordan: A cross-sectional study
Source: PLoS One. 2023 Jun 1;18(6):e0286289. doi: 10.1371/journal.pone.0286289 (PMC10234543; doi:10.1371/journal.pone.0286289)
Supplement: Attachment — Submitted filename: Response to reviewers.docx [file pone.0286289.s007.docx]

**Title of the manuscript: COVID-19 vaccine hesitancy among Jordanian women who are planning to be pregnant, are pregnant, or are breastfeeding**

Dear all,

Thank you for your valuable comments, please see the table below. It provides point-to-point response to your comments. Thank you so much for taking time reading and providing comments to improve the manuscript.

| **Comments** | **Response** |
| --- | --- |
| **Editor’s comments (Journal requirements)** |  |
| 1. Please ensure that your manuscript meets PLOS ONE's style requirements, including those for file naming | Fixed |
| 2. We note that the grant information you provided in the ‘Funding Information’ and ‘Financial Disclosure’ sections do not match | Fixed; the funding information is now provided in the cover letter |
| 3. Financial statement | Edited |
| 4. Please remove any funding-related text from the manuscript and let us know how you would like to update your Funding Statement. | Changed and funding information is now provided in the cover letter |
| 5. Data availability | Uploaded |
| **Reviewer’s comments** |  |
| Abstract  You have to consider that this is a new pandemic and little evidence is available. | A statement was added accordingly |
| Section 2.3  could you please verify or elaborate what do you mean by other women. | Clarified |
| Methods: replace the Arabic reference | Replaced. Samannodi, 2021 is the Arabic one |
| Ethical consideration: IRB number | The ASU IRB was added and there is no other IRBs, it was only access that was granted from other organisations. |
| You have stated that “This questionnaire has been previously used in studies around the globe, and has been 7 reported to be a valid and reliable questionnaire to be used in Arabic communities (Lin et al., 2020, Samannodi, 2021). | Corrected and clarified in the text. Lin is the Chinese reference and Samannodi is the Arabic one. |
| Data collection: make a justification  Why you will find breast feeding women and those who are planning to get pregnant in maternity clinics. Do not you think this is a limited site for recruitment | Justification was added in the text |
| You did anova and I was expected to see posthoc to include other women | The table now includes the ANOVA and post-hoc |
| Table 1 | Done |
| Titles of tables | Corrected |
| Results | Corrected |
| I was expected to see multiple regression analysis at the end. Why you stopped your data at bivariate Pearson’s correlations. I think a stronger prediction model will be good addition | I agree, Regression would be better analysis test but due to the nature of the variables ANOVA and post-hoc worked good. |
| Discussion  The current study showed that women who were married, pregnant women, planning to be pregnant, or breastfeeding women had higher COVID-19 vaccination hesitancy compared to other women.  Why you added the word married. It is a given fact | Some might be divorced or widowed ? and in other cultures women might be pregnant without being legally/civil marriage. |
