# Supplementary material for: COVID-19 vaccine hesitancy among women planning for pregnancy, pregnant or breastfeeding mothers in Jordan: A cross-sectional study
Source: PLoS One. 2023 Jun 1;18(6):e0286289. doi: 10.1371/journal.pone.0286289 (PMC10234543; doi:10.1371/journal.pone.0286289)
Supplement: Attachment — Submitted filename: Response to Reviewers.docx [file pone.0286289.s008.docx]

**Title**: COVID-19 Vaccine Hesitancy Among Women Planning for Pregnancy, Pregnant or Breastfeeding in Jordan

Dear all, thank you for your comments, please see the table below providing the response for your valuable comments that I am sure it added to the study.

| **Comments** | **Response** |
| --- | --- |
| **Editor’s comments** | |
| Please ensure that your manuscript meets PLOS ONE's style requirements, including those for file naming. | Fixed accordingly  References were also fixed |
| The title is not scientifically written. It needs to be revised. | Changed |
| ABSTRACT: It is too long; it needs to be shortened | Shortened |
| INTRODUCTION: The text of the introduction is too long with redundancy and diffusion. | Shortened |
| Newest Global/ Regional/ Jordan prevalence of COVID-19 vaccine hesitancy among pregnant and breastfed Jordanian women should be stated. | Added to the second last paragraph of the introduction |
| Risk factors associated with COVID-19 vaccine hesitancy among pregnant and breastfed Jordanian women should be clearly stated. | Added to the second last paragraph of the introduction |
| The current situation of other developed and developing countries should also be added. | Mentioned in the second last paragraph of the introduction |
| Explaining why this topic was chosen for analysis in this article is not well written. The benefits of conducting the study to the community should be explained. | Added to the last paragraph in the introduction |
| The aim should be clearly stated. | Stated at the end of introduction |
| METHODS: The characteristics of the study participants should be mentioned as inclusion criteria and exclusion criteria. | Stated in section 2.3 |
| Definitions as pregnant woman, women planning to be pregnant, breastfed woman, ……. etc. should be mentioned. | Added to section 2.3 |
| It is advisable to include the questions as per each domain in the methodology. How long did it take to complete the questionnaire(s)? Mention the number of questions in the questionnaire(s). As well as did the participant answer it themselves or with assistance from researcher? | Done see Sections:  2.6  2.4.1.  2.4.2 |
| Since you conducted the data collection with online means, what strategy was devised to increase the accuracy of the study and the accuracy of the answers? What types of technique the authors used to keep/control the data quality? | Corrected in section 2.6 |
| RESULTS: Titles of the tables has to be improved to be more specific. | Corrected  The title of tables will be so long when the are more specific |
| The correlation between perceptions of the seriousness of COVID-19, perceived benefits, motivation and causes of action of being vaccinated, and hesitancy should be added to the comments of table 4. | Done |
| It is advisable to add multiple regression analysis at the end of the results. | There was no need to use Multiple regression as the study did not discuss predictors but discussed the differences between women pregnancy status and their hesitancy to receive the vaccine |
| DISCUSSION: Please start the discussion with the study objective, a short summary of the study, and the main findings. | Added |
| Discuss by using the scientific reasoning the COVID-19 vaccine hesitancy among pregnant and breastfed Jordanian women in other developing and developed countries with similar context. The manuscript could be greatly strengthened if the authors could compare the findings of the study with other findings and state the reasons for the strengths and weaknesses in each section. | This was done in the discussion section. The second paragraph discuss our result with other contexts (developing and developed) and after that each section discusses each piece of results in relation to other studies. |
| CONCLUSION: It should be specific and based on the findings of the study. | It is specific to the results of our study |
| STRENGTHS AND LIMITATIONS: Please analyze the strengths of the study | Added |
| Rewrite the limitations of the study | Done |
| **Reviewer’s 1 comments** | |
| Introduction  1. The introduction seems to be long (2 pages). The authors should consider making short and concise (introduce the topic, what is already known, what is unknown/justification for the study and finally aim/objective) | Shortened |
| Methods  Study design  2. The authors mentions that it was “correlational” study design. Why do they refer the study that way? In correlational studies, the study unit is not people but geographical. | Sure, the it is a not correlational and this was fixed in the manuscript. |
| 3. Some information is missing for sample size calculation. Consult a Statistician for the sample size calculation for cross-sectional studies with a comparison group. Some assumptions especially the two proportions (p1 and p2) and there references are missing. | Corrected by adding the effect size to the equation and the reference as well |
| 3. “…..women who are planning to be pregnant, pregnant women, and breastfeeding women, and to compare them with other women”. Identifying pregnant and breastfeeding women. What was the distinction between women planning to be pregnant and other women? How were women planning to be pregnancy identified? There is a danger of misclassification. | Clarification was added to section 2.3 |
| 4. Please include the name of ethical institution and the IRB number | Added |
| 5. How was the barcode/ link for the survey distributed to the participants? Was the scanning done via a mobile phone, bar code scanner etc? Please specify these details sequentially. Was measures were put in place that each participate answers the questionnaire once. What measures were also put to make sure those outside the study area did not participate? With social media link can be easily shared. | See section 2.6 |
| Results  6. The sample size calculation assumptions were to compare the outcomes of women who are planning to be pregnant, pregnant women, and breastfeeding women with the “other women”. The “other women” are not coming out in the results showing the comparison |  |
| 7. The calculated sample size was 847 but you interviewed more than double 1, 874. Any implications and if yes these should be discussed. | No. It is stated Table 1 then comma, then the actual number of participants, which was 874. However, we re-wrote the sentence so it doesn’t make the same confusion. |
| 8. In Table 1, were age and years of education normally distributed? The mean and SD is reported for normally distributed data, otherwise report the median. | Yes I checked them and they were normally distributed as the skew index for both was less than 2 standard error |
| 9. Results section 3.2, “In this study, perceptions of COVID-19 seriousness, vaccine hesitancy, perceived benefits, and motivation and causes of action to take the vaccine were all considered as dependent variables whereas other variables were grouping variables”. This should be moved to the methods sections on variables and expanded further. Consider using the term outcome(s) and independent (exposure, explanatory) variables which easier to follow. | Moved and corredcted |
| Discussion  10. The first paragraph of the discussion should summarise the main findings of the study and later paragraphs should discuss finding by finding | Added |
| 11. Include strengths of the study | Added |
| 12. What is listed as limitations are not limitations. Usually limitations should include methodological issues which could have affected the validity of the results. Please revise | Revised |
| 13. The authors should maintain the sequence “Women planning to become pregnant, who are pregnant, and who are breastfeeding” throughout the paper. In some instances it not followed. | Corrected throughout the manuscript |
| **Reviewer’s 2 comments** | |
| 1. Study design: The researchers only indicated that an online cross-sectional correlational study was conducted without enough information as what that study design entails and why that type of study was chosen over study designs. It will beneficial to elaborate more on why you chose that design and what constitute cross-sectional correlational study. Related to the above statement is the lack of description of what G-power software is for the calculation of sample size. Please, provide brief explanation of the software. | The designs was corrected accordingly and the sample size calculation was also corrected all over the study. |
| 2. Ethical consideration: Please, include the ethical approval number and the name of the institution that granted the approval instead of just stating that the institution of the lead researcher. This is section 2.5. | Added in the ethics section |
| 3. Dependent and independent variables: There appears to be an issue of clarity in terms of which variables are dependent and which are independent. You have lumped them up in my opinion. The dependent variable I see here is COVID-19 vaccine hesitancy which in other words not getting vaccinated. The other variables are independent variables in my opinion which are supposed to impact the uptake or otherwise of the dependent variable hesitancy. Please, clarify. | A paragraph was added to section 2.4 under the variables to clarify this issue |
| 4. I think including history of chronic diseases in the results section detracts from the main objective of the study and has the potential to the focus of the readers from the most important variables in the study, that is the key concepts of health believe model and its impact on covid vaccine uptake among the women in this study. | Don’t know what you mean |
| 5. A very important recommendation I have for you is to get professional editing of the manuscript done. Though not the vaccine of the study or my review, there are inconsistencies in the tenses of the sentences as some appear to present terms and others in the past tense. Since the study is completed and this manuscript is a report of the results, it will be appropriate to ensure that everything is written in past tense unless it is a description of something. | Considered all over the manuscript |
