# Supplementary material for: COVID-19 vaccine hesitancy among women planning for pregnancy, pregnant or breastfeeding mothers in Jordan: A cross-sectional study
Source: PLoS One. 2023 Jun 1;18(6):e0286289. doi: 10.1371/journal.pone.0286289 (PMC10234543; doi:10.1371/journal.pone.0286289)
Supplement: Attachment — Submitted filename: PONE-D-22-08549R2-1.docx [file pone.0286289.s009.docx]

**Comments:**

**Title**

- Could you please add the study design to the title?

**Abstract**

- The following sentence needs better explanation: "Married women, those who were previously infected with COVID-19, and those who had chronic diseases reported statistically significant lower levels of perception, perceived benefits, motivation, and causes of action, and significantly higher levels of hesitancy than unmarried women". Perception, perceived benefits, Motivation for what? And hesitancy against what?

- "Women who were planning for pregnancy, pregnant, or breastfeeding in Jordan were relatively hesitant to take COVID-19 vaccine". This is a vague statement. When you suggest "relatively hesitant", relative to what? Could you please be more concrete?
- "Women who were planning for pregnancy, pregnant, or breastfeeding in Jordan were relatively hesitant to take COVID-19 vaccine". Please delete the second were in the sentence.
- "Therefore". Please add a full stop before "Therefore."

**Introduction:**

- "A study in the UK of 472 pregnant women diagnosed with COVID-19 and admitted to…." Admitted to what?
- "womenplanning" please separate the two words.
- "Therefore, this study aims to investigate vaccine hesitancy among these groups in comparison with other women in the national context". Your study's aim or research question does not match your chosen cross-sectional design. It implies that you are interested in comparing the outcomes of interest between two groups with different exposures. I suggest replacing this statement with what you have mentioned in other parts of your paper: "The aim of this study was to investigate COVID-19 vaccine hesitancy among women who are planning for pregnancy, currently pregnant, and breastfeeding women in Jordan." This is more appropriate for the cross-sectional design of your study.

**Methods**

- Data is plural. Please consider this throughout the text.
- Why didn't you include illiterate women? Could you explain this please?
- Regarding the sample size calculation, have you considered the anticipated incidence of vaccine hesitancy reported in other similar studies in your calculation?
- How many maternal clinics were involved in this study?
- "breasfeeding" should be replaced with "breastfeeding”.
- "However, the study included two groups of women with sub-groups (i.e. the first group: those who were planning for pregnancy: women trying to get pregnant or thinking of it, pregnant women: with positive signs of pregnancy, or breastfeeding women: women breasfeeding their children exclusively or not regardless the age of the child. The second group: women who do not fall into the first group categories (planning to be pregnant, pregnant or breastfeeding))." This isn't very clear. Please clearly define the main groups and what the subgroups are.
- Why did you use convenient sampling but not random sampling? Could you explain this, please?
- Regarding this statement: "make sure that a representative number of women who are planning for pregnancy, pregnant, or breastfeeding were included." Given that you did not apply random sampling in your study, increasing the number of participants you recruit for your study would not "ensure" that your sample would be representative of your target population. You cannot claim that your sample represents your target population based on the non-random sampling methods you used. Instead, you could imply that by recruiting a higher number of participants for your study, you increase the likelihood that your sample could represent your target population.
- Under "Demographic characteristics sheet", you mentioned asking the participants about their "residency"! Do you mean here their residency status, address of residency, or something else? Please clarify this.
- This statement is inaccurate: "An IT professional designed the barcode in a way that allowed participants to answer the questionnaire only once." Do you mean that the questionnaire cannot be answered from the same device, "smartphone", more than once?
- This statement is inaccurate: "To ensure….. the accuracy of the answers, the link provided to the participants allows submission only when all answers are completed." Do you mean: to avoid missing data?
- Statistical analysis: You used parametric methods: This should be applied to normally distributed data. Please confirm this. If the data were non-normally distributed, please apply the appropriate methods.
- Have you conducted a pilot study before conducting your actual study?

**Results**

- The final part of Table 1 (Perception of seriousness of COVID-19, Vaccine hesitancy, Perceived benefits of vaccine, and Motivation and cause of action of taking vaccine) is unclear. Please illustrate these results as figures.
- It seems that you have applied Pearson r correlation test. This was not mentioned in the methods parts. It has to be mentioned under the statistical analyses part. You should also explain how the data you used in your analyses satisfied this test's assumptions. Otherwise, using the Pearson r correlation test could be inappropriate.
- Is there any lessons or messages for policy, practice or research based on the outcomes of this study? Please clearly state them.

**Discussion**

- Your study has excluded illiterate women, women who do not have devices “smartphones” to access your questionnaire, women who have no access to the internet, and maybe other groups of women! Please reflect on what this means for the outcome of your study.
- Strengths and limitations: The study has more limitations to be reported. These limitations include: Using convenient sampling (instead of random sampling); including only literate women who have access to the internet.
