# Supplementary material for: COVID-19 vaccine hesitancy among women planning for pregnancy, pregnant or breastfeeding mothers in Jordan: A cross-sectional study
Source: PLoS One. 2023 Jun 1;18(6):e0286289. doi: 10.1371/journal.pone.0286289 (PMC10234543; doi:10.1371/journal.pone.0286289)
Supplement: Attachment — Submitted filename: ResponseToReviewers.docx [file pone.0286289.s010.docx]

**Title: COVID-19 Vaccine Hesitancy Among Women Planning for Pregnancy, Pregnant or Breastfeeding in Jordan: A Cross-sectional Study**

**Many thanks for your valuable time and comments. The research team found these comments are contributing to the study. Thanks again for taking time reviewing the paper, please see our response below (in blue)**

**.**

**Title**

Could you please add the study design to the title?

- Added

**Abstract**

The following sentence needs better explanation: "Married women, those who were previously infected with COVID-19, and those who had chronic diseases reported statistically significant lower levels of perception, perceived benefits, motivation, and causes of action, and significantly higher levels of hesitancy than unmarried women". Perception, perceived benefits, Motivation for what? And hesitancy against what?

- Corrected, please

"Women who were planning for pregnancy, pregnant, or breastfeeding in Jordan were relatively hesitant to take COVID-19 vaccine". This is a vague statement. When you suggest "relatively hesitant", relative to what? Could you please be more concrete?

- Clarified

"Women who were planning for pregnancy, pregnant, or breastfeeding in Jordan were relatively hesitant to take COVID-19 vaccine".

- Deleted

"Therefore". Please add a full stop before "Therefore."

- Added

**Introduction:**

"A study in the UK of 472 pregnant women diagnosed with COVID-19 and admitted to…." Admitted to what?

- Clarified

"womenplanning" please separate the two words.

- Done

"Therefore, this study aims to investigate vaccine hesitancy among these groups in comparison with other women in the national context". Your study's aim or research question does not match your chosen cross-sectional design. It implies that you are interested in comparing the outcomes of interest between two groups with different exposures. I suggest replacing this statement with what you have mentioned in other parts of your paper: "The aim of this study was to investigate COVID-19 vaccine hesitancy among women who are planning for pregnancy, currently pregnant, and breastfeeding women in Jordan." This is more appropriate for the cross-sectional design of your study.

- Considered

**Methods**

Data is plural. Please consider this throughout the text.

- Considered

Why didn't you include illiterate women? Could you explain this please?

- The illiteracy rate for this age group in Jordan is lower than 1% and the data collection procedure was online. So, the researchers wanted women to answer the questionnaire without having to be physically with them especially during the pandemic of COVID19. This facilitated the data collection process

Regarding the sample size calculation, have you considered the anticipated incidence of vaccine hesitancy reported in other similar studies in your calculation?

- Well, we tried to make the calculation based on the previous studies but the G-power would not allow us for such an option. So, we aimed for a large representative sample size.

How many maternal clinics were involved in this study?

- Twelve and this was added to the manuscript

"breasfeeding" should be replaced with "breastfeeding”.

- Replaced

"However, the study included two groups of women with sub-groups (i.e. the first group: those who were planning for pregnancy: women trying to get pregnant or thinking of it, pregnant women: with positive signs of pregnancy, or breastfeeding women: women breasfeeding their children exclusively or not regardless the age of the child. The second group: women who do not fall into the first group categories (planning to be pregnant, pregnant or breastfeeding))." This isn't very clear. Please clearly define the main groups and what the subgroups are.

- Clarified

Why did you use convenient sampling but not random sampling? Could you explain this, please?

- We acknowledge that random sampling might be better but convivence sampling was used due to the geographical proximity, availability in a given time and easiest for the researcher.

Regarding this statement: "make sure that a representative number of women who are planning for pregnancy, pregnant, or breastfeeding were included." Given that you did not apply random sampling in your study, increasing the number of participants you recruit for your study would not "ensure" that your sample would be representative of your target population. You cannot claim that your sample represents your target population based on the non-random sampling methods you used. Instead, you could imply that by recruiting a higher number of participants for your study, you increase the likelihood that your sample could represent your target population.

- Fixed

Under "Demographic characteristics sheet", you mentioned asking the participants about their "residency"! Do you mean here their residency status, address of residency, or something else? Please clarify this.

- Clarified

This statement is inaccurate: "An IT professional designed the barcode in a way that allowed participants to answer the questionnaire only once." Do you mean that the questionnaire cannot be answered from the same device, "smartphone", more than once?

- Yes

This statement is inaccurate: "To ensure….. the accuracy of the answers, the link provided to the participants allows submission only when all answers are completed." Do you mean: to avoid missing data?

- Clarified

Statistical analysis: You used parametric methods: This should be applied to normally distributed data. Please confirm this. If the data were non-normally distributed, please apply the appropriate methods.

- This is now clarified in the text and all variables included in the inferential analysis were normally distributed.

Have you conducted a pilot study before conducting your actual study?

- We piloted the material and for 10% of the sample size and all questions were clear and there was no modification to the process/instruments. Therefore, those were included in the final analysis. A sentence was added to the text clarifying this issue.

**Results**

The final part of Table 1 (Perception of seriousness of COVID-19, Vaccine hesitancy, Perceived benefits of vaccine, and Motivation and cause of action of taking vaccine) is unclear. Please illustrate these results as figures.

- We split the table and all tables were numbered accordingly as suggested.

It seems that you have applied Pearson r correlation test. This was not mentioned in the methods parts. It has to be mentioned under the statistical analyses part. You should also explain how the data you used in your analyses satisfied this test's assumptions. Otherwise, using the Pearson r correlation test could be inappropriate.

- Corrected and it is now clarified in the statistical part section.

Is there any lessons or messages for policy, practice or research based on the outcomes of this study? Please clearly state them.

- Added to the discussion section

**Discussion**

Your study has excluded illiterate women, women who do not have devices “smartphones” to access your questionnaire, women who have no access to the internet, and maybe other groups of women! Please reflect on what this means for the outcome of your study.

- This is really an important comment. A paragraph was added to reflect on this this on the discussion and the methods.

Strengths and limitations: The study has more limitations to be reported. These limitations include: Using convenient sampling (instead of random sampling); including only literate women who have access to the internet.

- Added

**Other comments from the email**

**Abstract**: It had 419 wording which was too much as the PLOS guidelines. Nothing mentioned in abstract about the questionnaire used in this study. P-values for statistically significant should reported. Abstract should be revised, and only relevant important findings should be reported.

🡪 Revised and shortened

**Background**:

Pleas change this heading to Introduction.

🡪 Changed

The statement “The Centers for Disease Control and Prevention (CDC) reported that all individuals at any age can be infected with the virus and their infection can be severe; however, individuals with underlying medical conditions are at higher risk of having more severe consequences of COVID-19, [2, 5].” Reference 2 was not published by CDC.

🡪 Removed

The authors stated that “ Several COVID-19 vaccines (hereinafter “vaccines”) were developed in an attempt to end the pandemic, four of which received emergency use authorization approval to be used in Jordan, namely: Pfizer/ BioNTech, Sputnik V, Oxford/ AstraZeneca, and Sinopharm [2, 11].” Both references 2 and 11 were about frequently asked questions on COVID-19, nothing about vaccines.

🡪 Corrected

The statement “…availability and accessibility of the vaccine (Limaye et al., 2022)” and “..breastfeeding women (Bianchi et al., 2022)”. For both number of the reference should be used rather than the name of the authors.

- Corrected

The authors stated that “This hesitancy was explained by two main reasons.” Which contradict with their statement at the end of the page “Several factors can affect vaccine hesitancy”,

🡪 that true, two MAIN reasons and several factors associated with hesitancy.

The authors stated that “To our knowledge, vaccine hesitancy among women planning for pregnancy, pregnant and breastfeeding was not studied before in Jordan.” This was an assumption as authors were unaware of unpublished work in Jordan in this regard.

Therefore, the introduction (background) of this manuscript should be revised. 🡪 Revised

**Methodology**: This section requires major revision.

The questionnaire used in this study had major issues.

The authors stated that “This study implements an online cross-sectional study.” Since the data collection occurred during the COVID-19 pandemic , was it a virtual survey and where was it (home or clinic).

- Participant visited the clinics for they regular check up and this is where they scanned the barcode and had access to study but when they filled in the questionnaire the might be anywhere.

What were the exclusion criteria? How many women were excluded?

- In this study, 877 accessed the questionnaires and 847 fully competed the study.

The reasons to increase sample size were not convincing.

🡪 Justified

Under demographic characteristics sheet “what were the definitions of comfortable, varies and tight of financial status?

- According to the nationally studies, people have an income less than 500 JOD were tight, between 500-800 were varies and above that comfortable.

The authors stated that “The Arabic Version of Perceptions, Hesitancy, Perceived Benefits, Motivation and Cause of Action About COVID-19 Vaccination Questionnaire This questionnaire has been previously used in studies around the globe (Lin et al., 2020), and has been reported to be a valid and a reliable questionnaire to be used in Arabic communities [16, 21].” Both references did not have the Arabic questionnaire .

Reference 16. Samannodi, M., COVID-19 Vaccine Acceptability Among Women Who are Pregnant or Planning for Pregnancy in Saudi Arabia: A Cross-Sectional Study. Patient Preference and Adherence, 2021. Volume 15: p. 2609-2618. While 21. Lin, Y., et al., Understanding COVID-19 vaccine demand and hesitancy: A nationwide online survey in China. 2020. 14(12): p. e0008961.

Indeed reference 16 used questionnaire published by Almaghaslah D, Alsayari A, Kandasamy G, et al. COVID-19 vaccine hesitancy among young adults in Saudi Arabia: a cross-sectional web-based study. Vaccines. 2021;9(4):330. doi: 10.3390/vaccines9040330.

Almaghaslah D, et al developed the questionnaire on previous systematic review on work published by Lin C, Tu P, Beitsch L. Confidence and receptivity for COVID-19 vaccines: a rapid systematic review. Vaccines 2020;9(1):16. doi: 10.3390/vaccines9010016.

Therefore, the questionnaire used this study should be based on Almaghaslah D, et al work.

- The references have been corrected in this paragraph and Almaghaslah D, et al. was added

The validity of the questionnaires used in this study, and their Cron.bach’s alpha and reliability scores should be reported. The questionnaire should be explained in detail. How many sections in the questionnaire ( Reference 16 had 5 sections while authors reported 4 domain) ? the possible scores for each section / domain must be calculated?

The authors stated “Regarding the second section (participants’ perceptions about COVID-19), the higher the score the weaker the perception about the seriousness of the COVID-19 situation. Regarding the third section (participants’ hesitancy about receiving the COVID-19 vaccine), the higher the score the greater the hesitancy to receive the vaccine. Regarding the fourth section (participants’ perceived benefits of receiving the COVID-19 vaccine), the higher the score the stronger the perception about the perceived benefits of the vaccine. Regarding the last section (participants’ motivations and causes of action for receiving the COVID-19 vaccine), the higher the score the lower the motivation or likelihood to take action to receive the vaccine”. This contradicts with the findings reported in reference 16.

- A sentence was added to the tool section represents the Chronbachs alpha for this study.
- It is correct that the original tool has five sections (i.e. the first section presented the demographics of the participants which was added to the other four sections). In this study, the demographic section was presented in a separate section (i.e. the Demographic characteristics sheet) while the questionnaire has the other four sections same to the original one. So, it is the same questionnaire.
- Regarding the interpretation for each section, it is the same as in Almaghaslah D and Samannodi, M.,

What was the sampling technique? 🡪 Stated in section 1.3

**Results**:

The whole results should be revised once the comments in methodology were incorporated.

Tables: Table 1 was very busy and did not fit in one page. I suggest either to split it or delete unimportant variables in this table.

- The interpretation of the questionnaire and the table was corrected accordingly. All tables were corrected accordingly.

Under Table 1, What was the definition of other women?

- Other women, those who were no pregnant/planning for pregnancy, not breast feeding

Under Table 1, the total married women was 492 and this contradict with the total women (428) who were pregnant , planning for pregnancy and breastfeeding. Please revise.

- 492 married, of those 428 who were pregnant , planning for pregnancy and breastfeeding.

Table 2: It was very busy and did not fit in one page. I suggest either to split it or delete unimportant variables in this table.

- We could not minimize this table as post hoc was important to be presented.

The data for “However, the results showed no significant difference in perception, hesitancy, perceived benefits, and motivation and causes of action of women according to their residency and financial status” were not shown in Table 2.

- As these were not significant, so it was suggested not to include them to minimize the table but it was important to state that these were examined but no significant results were found.

I suggest to delete Table 2 and replace it with another Table to calculate the total mean score for each domain of pregnant , planning for pregnancy and breastfeeding and compare it to the total mean score of other women.

Table 3: It was very busy and did not fit in one page. I suggest either to split it or delete unimportant variables in this table. Furthermore, it was inappropriate statistical analysis.

Authors must do statistical analysis for the mean vaccination perception, hesitancy, perceived benefit and cause of action score stratified by demographic characteristics for women Who are pregnant or planning to be or breastfeeding in order to adjust for all possible confounding factors.

- Exactly, this table represents the mean vaccination perception, hesitancy, perceived benefit and cause of action score stratified by demographic characteristics for women Who are pregnant or planning to be or breastfeeding

**Limitations of the study**: It should be explained in details and logical ways. Limitations section should be focused on biases, such as recruitment, personal, recall, and misinterpretation biases as these were possibilities in this study.

🡪 Revised

General comments: The manuscript was full of typo and in dire need to be edited by someone more proficient in English.

🡪 Checked
