# Supplementary material for: COVID-19 vaccine hesitancy among women planning for pregnancy, pregnant or breastfeeding mothers in Jordan: A cross-sectional study
Source: PLoS One. 2023 Jun 1;18(6):e0286289. doi: 10.1371/journal.pone.0286289 (PMC10234543; doi:10.1371/journal.pone.0286289)
Supplement: Attachment — Submitted filename: ResponseToReviewers.docx [file pone.0286289.s011.docx]

**Title: COVID-19 Vaccine Hesitancy Among Women Planning for Pregnancy, Pregnant or Breastfeeding Mothers in Jordan: A Cross-sectional Study**

Many thanks for your valuable comments reviewing this manuscript many times. The research team agree with your input and found these comments are contributing to the study; please see our response below (in blue)

.

**Journal Requirements:**
Please review your reference list to ensure that it is complete and correct. If you have cited papers that have been retracted, please include the rationale for doing so in the manuscript text, or remove these references and replace them with relevant current references. Any changes to the reference list should be mentioned in the rebuttal letter that accompanies your revised manuscript. If you need to cite a retracted article, indicate the article’s retracted status in the References list and also include a citation and full reference for the retraction notice.

Authors’ response:

Referecnes were checked, no retracted were cited in the study

**Additional Editor Comments:**

¬Titles of the tables has to be improved to be more specific.

Authors’ response:

Titles of the tables have been changed

**Reviewer #5:**

After reading reading this manuscript many times and reviewing it for three times, I maintain my review conclusion that the authors have done remarkable job of working hard to improve the entirety of the manuscript from my observation. The methodology, statistical analysis, and discussions are very well developed and presented. Again, the subject matter of this manuscript is important for maternal and child health as well as other important diseases/conditions of public health importance that require vaccine interventions.

Authors’ response:

Thank you for your time reading the mansucript many times and it wouldnot be that good without your contribution and comments.

**Reviewer #8:**

Dear authors,
Thank you for addressing my comments in your revised manuscript. I recommend publishing your manuscript.

Authors’ response:

Thank you for your recommendation
